# The effect of authentic leadership and organizational climate on job embeddedness in Chinese nurses: the mediating role of affective commitment

**DOI:** 10.3389/fpsyg.2025.1572684

**Published:** 2025-10-01

**Authors:** Xiang Zhou, Xue Bai, Xin Yang, Yan Chen

**Affiliations:** ^1^School of Sociology and Political Science, Shanghai University, Shanghai, China; ^2^Department of Critical Care Medicine, Shandong Provincial Hospital Affiliated to Shandong First Medical University, Jinan, China; ^3^Department of Nursing, The First Affiliated Hospital of Henan University of Traditional Chinese Medicine, Zhengzhou, China

**Keywords:** authentic leadership, organizational climate, affective commitment, job embeddedness, nurses

## Abstract

**Introduction:**

Job embeddedness is beneficial for promoting nurse retention, positive organizational behavior, and patient outcomes. Therefore, it is crucial to give more attention to the job embeddedness of nurses. This study aims to explore the mediating role of affective commitment in the perception of authentic leadership, organizational climate, and job embeddedness among Chinese nurses.

**Methods:**

This cross-sectional study surveyed 716 clinical nurses from hospitals in northern China. The data analysis methods included a descriptive statistical analysis, Pearson correlation analysis, regression analysis, and Bootstrap mediation effect test.

**Results:**

We found a significant positive correlation between authentic leadership, organizational climate, affective commitment, and the job embeddedness of nurses. Affective commitment partially mediates authentic leadership and job embeddedness and fully mediates organizational climate and job embeddedness.

**Discussion:**

Authentic leadership style is an important factor affecting the job embeddedness of nurses; affective commitment as a mediating variable more clearly reveals the impact of authentic leadership and organizational climate on job embeddedness. Nursing managers should develop an authentic leadership style, improve organizational climate, effectively enhance affective commitment, strengthen nurses’ job embeddedness, and promote nurse retention.

## Introduction

China has entered an aging society with the rapid growth of the elderly population and the improvement of public health literacy, which has led to an increase in the demand for medical and health services (Han et al., 2020; Li et al., 2022) and a significant increase in demand for nurses (Hu et al., 2022). However, the shortage of nursing staff has become a prominent issue faced by China (Min et al., 2018) and the world (World Health Organization [WHO], 2020). Although recruiting more nurses may be beneficial, a more effective approach may be to increase nurse retention (Kim and Park, 2023). This measure is related to significant training costs, provision of high-quality nursing services, and positive patient outcomes. Regarding employee retention, Mitchell et al. (2001) introduced the concept of job embeddedness, which reflects the sum of forces that limit people from leaving their current jobs. Compared to job satisfaction, organizational commitment, and job seeking, job embeddedness has shown a stronger explanatory power for employee turnover rates (Reitz and Anderson, 2011; Stroth, 2010). Moreover, previous studies have also confirmed the positive impact of job embeddedness on nurses’ behavior within the organization, such as voice behavior (Zhou et al., 2021), job performance (Song et al., 2024a), and innovative work behavior (Rahimnia et al., 2022). Therefore, it is of great significance to pay more attention to the job embeddedness of nurses.

### Authentic leadership and job embeddedness

Given the potential benefits of job embeddedness for nurse retention and organizational behavior, previous research on the antecedents of this topic has been increasing, including personal factors such as monthly income level (Song et al., 2024b), professionalism, and self-efficacy (Kim and Park, 2023). Furthermore, since job embeddedness is a construct composed of contextual and perceptual elements (Crossley et al., 2007), leadership styles and organizational environment have been proven to be important factors affecting employee embeddedness (Faraz et al., 2023; Nguyen et al., 2017; Oladeji and Ayinde, 2018). Among various leadership styles, transactional leadership, which is based on exchanges in the business context, received considerable attention in early research (Walumbwa and Wernsing, 2012). However, with increasing scholarly interest in humanistic perspectives, transformational leadership—which emphasizes recognizing individual needs and stimulating creative thinking to motivate people toward common goals—was proposed and developed (Bass, 1985; Martinez and Leija, 2023). Servant leadership, initially emerged as a branch of transformational leadership (Graham, 1991), differs notably in its focus: transformational leaders prioritize organizational objectives, whereas servant leaders prioritize followers, considering organizational goals a secondary outcome (Stone et al., 2004). Distinct from the aforementioned leadership styles, authentic leadership arose in response to the growing demand for more responsible, ethical, and authentic leadership approaches (Ladkin and Taylor, 2010). As a relatively emerging leadership style, authentic leadership moves away from the top-down perspective traditional in human resource management, instead adopting a dynamic view of the relationship between followers and leaders and co-creating a sense of authenticity through interaction (Gill et al., 2018). Consequently, authentic leadership is considered by some researchers to strengthen or even outperform other leadership styles in predicting group or organizational performance and organizational citizenship behavior (Avolio and Gardner, 2005; Banks et al., 2016).

Authentic leadership, rooted in positive psychology, is a leadership style based on the true self (Iszatt-White and Kempster, 2019). This leadership style has been listed as one of the six standards for creating and maintaining a healthy working environment by the American Association of Critical-Care Nurses (AACN). In cross-cultural contexts, authenticity is also recognized as a valued leadership attribute (Aponte-Moreno and Koulouris, 2017). Due to the inherent characteristics of authentic leadership—which include self-awareness, balanced processing, relational transparency, and the adoption of an internalized moral perspective—it can facilitate ethically grounded cultural adaptation (Vogelgesang Lester et al., 2009). This form of cross-cultural adaptability is further corroborated within the cross-cultural models proposed by Yang (2024), Khilji et al. (2015). Authentic leaders can act following the principles of honesty and integrity and their own values (Raso, 2019); can pay attention to the relationship with subordinates; and are good at identifying, cultivating, and developing the talents and abilities of subordinates in the management process (Demirtas, 2020). Social exchange theory (SET) addresses leader–member relationships formed through interactions and corresponding perceived obligations (Blau, 1986). According to SET, members under authentic leaders feel more support from leaders, have a deeper connection with leaders, and have a sense of obligation and higher self-efficacy. This sense of obligation encourages members to be more deeply involved in the job and make contributions in return for the support they feel (Demirtas, 2020; Nguyen et al., 2017), thus facilitating a higher degree of job embeddedness (Cho et al., 2019; Song et al., 2024b). Based on the above statements, we propose the following hypothesis:

Hypothesis 1: Authentic leadership has a direct impact on job embeddedness.

### Organizational climate and job embeddedness

Organizational climate refers to the common perception and feeling of organizational members toward the basic elements reflecting organizational cultural norms, attitudes, and values (Castro and Martins, 2010) This concept has been proven to positively or negatively impact employees’ attitudes and behaviors (Castro and Martins, 2010). Link, fit, and sacrifice are the three characteristics of job embeddedness, among which fit reflects employees’ perceived compatibility with the organizational environment (Mitchell et al., 2001). Hashim et al. (2015) found that employees who believe their personal values and goals align with the organization feel more comfortable with the organization. On the other hand, according to the SET, when employees perceive that they have gained more support from the organizational climate, they correspondingly develop a greater sense of responsibility toward the organization, and their embeddedness increases (Hashim et al., 2023). A study on young nurses found that a good organizational climate helps nurses develop a sense of belonging and irreplaceability to the hospital, which enhances their job embedding (Song et al., 2024a). Moreover, Hashim et al. (2015) found that organizational climate can shape positive employee behavior. Particularly, employees in an interactive and supportive organizational climate are more focused on the current organization, invest more energy into the organization, and deepen their integration with the organization (Hashim et al., 2015). Therefore, we propose the following hypothesis:

Hypothesis 2: Organizational climate has a direct impact on job embeddedness.

### Authentic leadership, affective commitment and job embeddedness

Affective commitment, as a dimension of organizational commitment, reflects employees’ emotional attachment, identification, and involvement in the organization (Meyer and Allen, 1991). Employees with strong affective commitment will continue to work in the organization and strive to contribute to the organization’s interests (Lilius et al., 2008). The authentic leadership model is based on the leaders’ moral character, integrity, and consistency of words and actions. In this leadership model, the leader can carry out high-quality communication with employees (Ribeiro et al., 2018), which not only deepens the emotional connection between the two parties but also the employees return leaders with more effective commitment (Paillé, 2009). Additionally, a previous study (Ribeiro et al., 2018) found that authentic leaders can form a sense of “we” with their subordinates, which strengthens the emotional bond between the entire team and enhances employees’ affective commitment. Studies have also verified the promoting effect of authentic leadership on affective commitment in a few employees of enterprises (Duarte et al., 2021; Ribeiro et al., 2018). Employees with high levels of commitment have a higher emotional attachment to the organization, which also means that they are more connected to the organization. Meyer et al. (2004) identified that employees with strong commitment integrate the norms and values of the organization, contribute to the effectiveness of the organization, and are keen to stay on the job, resulting in a higher degree of job embeddedness. Therefore, this study proposes the following hypothesis:

Hypothesis 3: Affective commitment plays a mediating role between authentic leadership and job embeddedness.

### Organizational climate, affective commitment and job embeddedness

As one of the main concepts of organizational behavior, organizational climate is a set of measurable attributes in the work environment that are perceived directly or indirectly by employees in the organization (Holloway, 2012). By providing employees with good working conditions and environment, supporting and assisting them in achieving personal goals, organizational climate helps to enhance employees’ level of commitment (including affective commitment) to the organization (Suliman and Iles, 2000). A study on lecturers in higher education institutions found that communication and career development, as two variables of organizational climate, can significantly predict affective commitment (Emmanuel et al., 2020). Furthermore, previous researchers considered organizational climate as a quantitative process of organizational culture that is determined by employees’ perceptions of the workplace, especially when employee values align with the organization, which can generate a stronger emotional attachment to the organization (Permarupan et al., 2013). Employees with higher emotional attachment to the organization are more willing to stay in the organization and can return to the organization with positive behaviors and attitudes, such as higher morale and productivity (Yaqub et al., 2021), which increase their engagement and sense of embeddedness in their job. Simultaneously, research on bank employees found that employees with high affective commitment exhibit higher loyalty to the organization (Bloemer and Odekerken-Schröder, 2006), which further encourages them to invest more time and energy into their jobs and deepens their connection with work. Thus, we hypothesized the following:

Hypothesis 4: Affective commitment plays a mediating role between organizational climate and job embeddedness.

### Hypothetical research model

**FIGURE 1:**
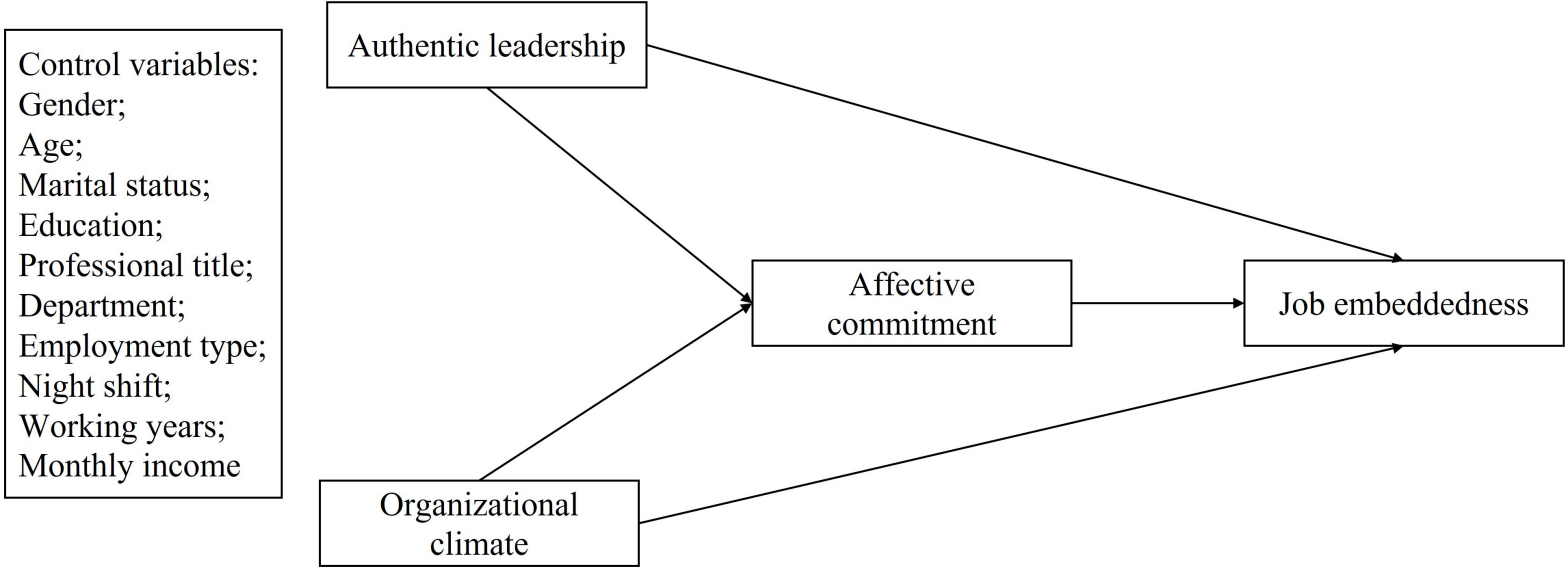
Hypothesized model.

Although some studies have explored the relationships between job embeddedness and authentic leadership (Cho et al., 2019; Erkutlu and Chafra, 2017; Song et al., 2024b) as well as organizational climate (Hashim et al., 2015, 2023; Song et al., 2024a), prior research has primarily focused on enterprise employees (Erkutlu and Chafra, 2017; Hashim et al., 2015), with limited attention given to newly graduated nurses (Song et al., 2024b) or younger nurses (Song et al., 2024a). Research on experienced nurses within the healthcare sector remains insufficient. As this group represents a critical and stable core of the nursing workforce, understanding and enhancing the factors that contribute to their job embeddedness is essential for developing effective retention strategies beyond the initial transition period. Second, some existing studies have utilized samples from countries such as South Korea (Cho et al., 2019), Malaysia (Hashim et al., 2023), and Turkey (Erkutlu and Chafra, 2017). Due to significant cultural differences, the findings of these studies may not be fully generalizable to Chinese society, which is characterized by collectivist values and high-power distance. Most importantly, existing research has primarily focused on examining bivariate correlations, such as the relationship between authentic leadership and job embeddedness (Song et al., 2024b), while lacking in-depth investigation into the joint mechanisms through which authentic leadership and organizational climate collectively influence job embeddedness. Leadership style and organizational environment are important factors affecting nurses’ attitudes and behaviors. This study analyzes the impact of authentic leadership and organizational climate on nurses’ job embeddedness from the perspectives of leaders and the organizational environment, as well as the mediating role of individual affective commitment, to provide ideas for the management and job embedding of experienced nursing staff. Figure 1 presents the conceptual framework of this study.

## Materials and methods

### Study design and participants

We conducted this cross-sectional study in Zhengzhou and Jinan, cities in northern China, from April to June 2023 using convenience sampling to investigate registered nurses with professional qualifications who had worked in general hospitals for more than 1 year. Before the investigation began, we explained the purpose and significance of the study in detail to the nursing manager of the hospital and contacted the head nurse of the department through the nursing manager. We secured the consent of each hospital nursing manager and sent a link to an online survey via WeChat to the clinical nurses after the regular department meeting. This link included the informed consent that the nurses had to confirm before taking the survey, notes for questionnaire filling, and the questionnaire. To prevent duplicate submissions, we set a limit of one response per IP address. Notably, the questionnaire was filled in anonymously, and participants had the right to withdraw from the survey if they felt uncomfortable with the survey questions. The data were monitored and recovered by two trained researchers. We obtained a total of 780 questionnaires in this study, and we selected 716 valid questionnaires after excluding incomplete questionnaires, those with the same answers for all items, those completed within 3 min, with an effective rate of 91.79%.

### Measures

#### Authentic leadership

In this study, a 16-item authentic leadership questionnaire was used to measure nurses’ perceived level of authentic leadership. Developed by Walumbwa et al. (2008), this questionnaire includes four dimensions, namely self-awareness, relational transparency, internalized moral perspective, and balanced processing. All items are rated on a Likert five-point scale, from 0 (not at all) to 4 (frequently). This tool has been widely used as an effective tool to measure authentic leadership (Alilyyani et al., 2022; Lee et al., 2019). Cronbach’s α coefficient in the current analysis was 0.958.

#### Organizational climate

Organizational climate was measured by the Gallup Q12 questionnaire. The tool consists of 12 items that are scored on a Likert five-point scale, with scores ranging from 1 (strongly disagree) to 5 (strongly agree). The questionnaire scores ranged from 12 to 60 points. The higher the score, the more positively the organizational climate is perceived by the individual. In a previous study on organizational climate, Gallup Q12 showed high effectiveness and reliability (Walker, 2012). The Cronbach’s α coefficient of this questionnaire was 0.922 in the current study.

#### Affective commitment

Affective commitment was assessed on a single-dimensional scale (Meyer et al., 1993). The scale consists of six items, such as “I would be very happy to spend the rest of my career with this organization.” A previous study on nurses showed that this scale is an effective assessment tool for affective commitment (Zhou et al., 2021). In this study, the Cronbach’s α coefficient of the scale was 0.923.

#### Job embeddedness

Nurses’ job embeddedness was measured using the global job embeddedness scale developed by Crossley et al. (2007). The scale consists of seven items, each of which is scored on a Likert five-point scale, from 1 (strongly disagree) to 5 (strongly agree). The total score on the scale is 7–35 points, and the higher the score, the stronger the degree of individual job embeddedness. This tool has been widely used to measure clinical nurse job embeddedness (Chen et al., 2024; Zhou et al., 2021). In the current analysis, the Cronbach’s α coefficient of the scale was 0.733, indicating acceptable reliability and validity.

#### Demographic variables

Demographic variables included participant gender (male vs. female), age (< 30, 30–39, ≥ 40), marital status (unmarried and married), education (junior college and below, undergraduate, master’s degree and above), employment type (authorized strength or contract employee), professional title (primary nurse, senior nurse, supervisor nurse, and deputy chief nurse and above), department (internal medicine, surgery, obstetrics and gynecology, pediatrics, emergency and ICU, and others), monthly night shift frequency (< 5, 5–8, > 8), working years (1–5, 6–10, 11–15, ≥ 16), and monthly income (≤ 3,000, 3,001–6,000, 6,001–10,000, ≥ 10,000).

### Statistical analysis

We performed a descriptive analysis of the participants’ demographic characteristics and study variables (authentic leadership, organizational climate, affective commitment, job embeddedness). We used Pearson’s correlation coefficient to analyze the correlation between the study variables. Additionally, we followed the regression analysis method recommended by Hayes (2017) to conduct the mediation effect analysis. Finally, we used random sampling to select 5,000 bootstrap samples from the current study’s data and calculated confidence intervals (CIs) to examine the mediating effect of affective commitment. If the upper and lower limits of the 95% confidence interval do not include 0, the indirect effect is considered significant (Preacher and Hayes, 2008). All data were analyzed using Stata MP 16.0.

## Results

### Descriptive and correlations analyses

Of the 716 participants, 96.65% were female participants. Table 1 presents the descriptive results of the demographic characteristics of the participants. Table 2 shows the mean, standard deviation, and correlation coefficient of the scores of the study variables in detail. Specifically, authentic leadership was positively correlated with organizational climate (*r* = 0.695, *p* < 0.001), affective commitment (*r* = 0.666, *p* < 0.001), and job embeddedness (*r* = 0.349, *p* < 0.001). Organizational climate was positively correlated with affective commitment (*r* = 0.763, *p* < 0.001) and job embeddedness (*r* = 0.364, *p* < 0.001). Affective commitment was positively correlated with job embeddedness (*r* = 0.414, *p* < 0.001).

**TABLE 1:** Demographics characteristics of participants (*N* = 716).

Variables	Category	Frequency (*N*)	Percentage (%)
Gender	Male	24	3.35
	Female	692	96.65
Age (years)	< 30	311	43.44
	30–39	336	46.93
	≥ 40	69	9.64
Marital status	Unmarried	189	26.4
	Married	527	73.6
Education	Junior college and below	122	17.04
	Undergraduate	570	79.61
	Master degree and above	24	3.35
Professional title	Primary nurse	76	10.61
	Senior nurse	336	46.93
	Supervisor nurse	281	39.25
	Deputy chief nurse and above	23	3.21
Department	Internal medicine	193	26.96
	Surgery	250	34.92
	Obstetrics and gynecology	83	11.59
	Pediatrics	63	8.80
	Emergency and ICU	63	8.80
	Others	64	8.94
Employment type	Authorized strength	167	23.32
	Contract employee	549	76.68
Night shift (times/month)	< 5	266	37.15
	5–8	310	43.40
	> 8	140	19.55
Working years	1–5	256	35.75
	6–10	242	33.80
	11–15	130	18.16
	≥ 16	88	12.29
Monthly income (¥)	≤ 3,000	55	7.68
	3,001–6,000	388	54.19
	6,001–10,000	201	28.07
	≥ 10,001	72	10.06

**TABLE 2:** Descriptive statistics and correlation coefficients between key variables (*N* = 716).

Variables	Mean	SD	1	2	3	4
Authentic leadership	44.71	10.69	1	–	–	–
Organizational climate	45.89	7.66	0.695***	1	–	–
Affective commitment	23.55	4.31	0.666***	0.763***	1	–
Job embeddedness	24.48	3.90	0.349***	0.364***	0.414***	1

***p < 0.001.

#### Mediation analysis

**FIGURE 2:**
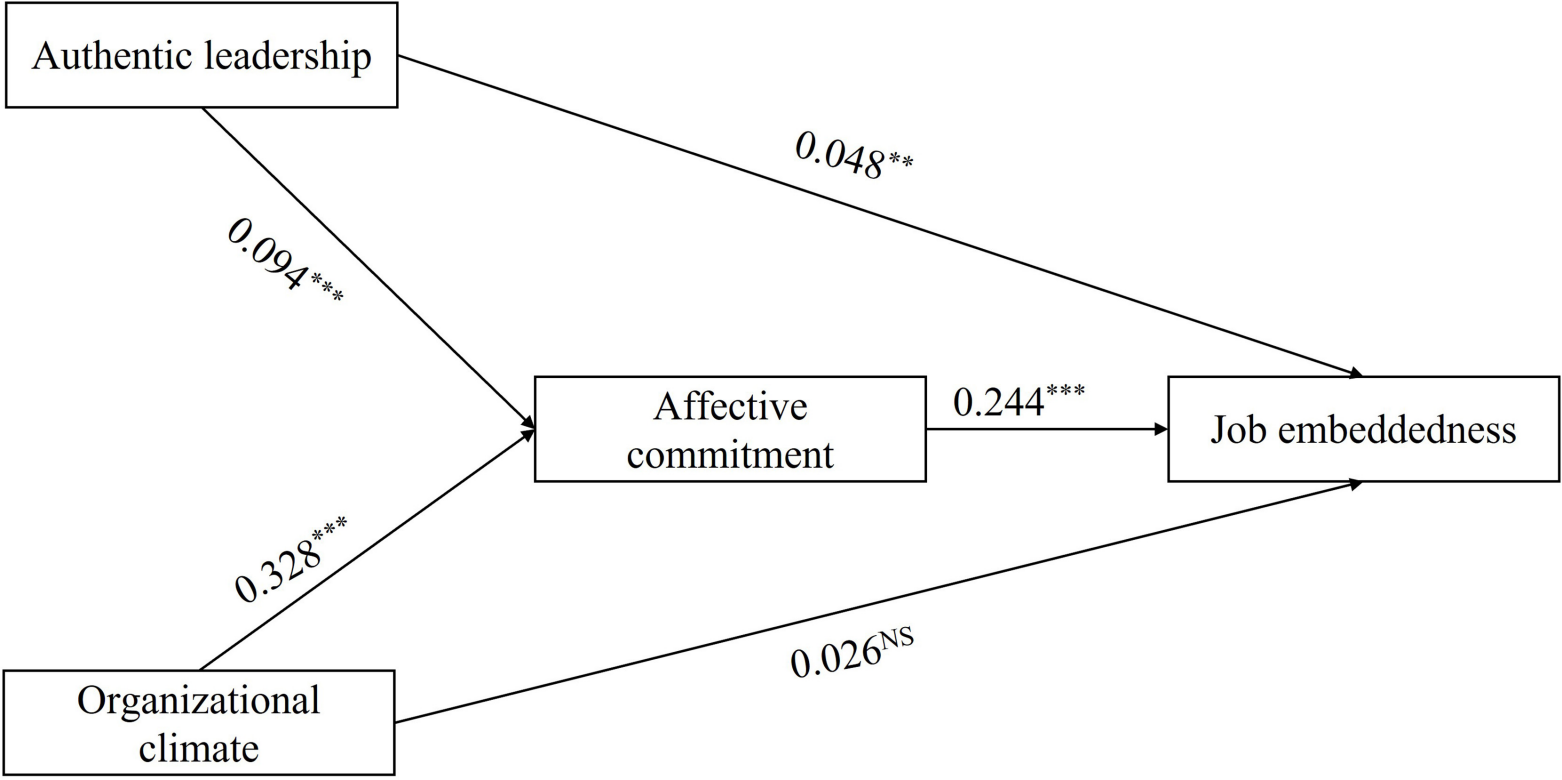
Result of the hypothetical model. No control variables are displayed. Ns, not significant. **p < 0.01, ***p < 0.001.

Table 3 shows the results of the regression analysis between variables. After controlling for the basic demographic characteristics of participants, the analysis found that authentic leadership (*b* = 0.048, *p* = 0.008) had a direct and significant impact on job embeddedness. Meanwhile, the impact of authentic leadership (*b* = 0.094, *p* < 0.001) on affective commitment was significant. Affective commitment (*b* = 0.244, *p* < 0.001) also had a significant impact on job embeddedness. However, the direct impact of organizational climate (*b* = 0.026, *p* = 0.379) on job embeddedness was not significant. The organizational climate (*b* = 0.328, *p* < 0.001) had a significant impact on affective commitment. Figure 2 presents the path coefficients between variables.

**TABLE 3:** Regression analysis of the influence of authentic leadership and organizational climate on job embeddedness among nurses in China.

Variables	Model 1: affective commitment				Model 2: job embeddedness			
	*b*	SE	*t*	*P*	*b*	SE	*t*	*P*
Authentic leadership	0.094	0.013	7.200	< 0.001	0.048	0.018	2.670	0.008
Organizational climate	0.328	0.018	18.130	< 0.001	0.026	0.029	0.880	0.379
Affective commitment	–	–	–	–	0.244	0.051	4.820	< 0.001
Gender (ref. female)	0.902	0.558	1.620	0.107	−0.188	0.744	−0.250	0.800
Age (30–39 ref. < 30)	0.499	0.304	1.640	0.101	0.291	0.405	0.720	0.472
Age (≥ 40 ref. < 30)	1.197	0.600	2.000	0.046	1.386	0.800	1.730	0.084
Married (ref. unmarried)	0.276	0.278	0.990	0.322	0.300	0.370	0.810	0.418
Education (undergraduate ref. Junior college and below)	−0.359	0.283	−1.270	0.206	−0.214	0.377	−0.570	0.572
Education (master degree or above ref. Junior college and below)	−2.934	0.610	−4.810	< 0.001	−0.189	0.825	−0.230	0.819
Employment type (contract employee ref. Authorized strength)	−0.452	0.282	−1.600	0.109	0.785	0.375	2.090	0.037
Professional title (senior nurse ref. primary nurse)	0.252	0.361	0.700	0.484	0.506	0.480	1.060	0.292
Professional title (supervisor nurse ref. primary nurse)	0.326	0.436	0.750	0.454	−0.254	0.580	−0.440	0.661
Professional title (deputy chief nurse and above ref. primary nurse)	0.659	0.778	0.850	0.398	−1.030	1.036	−0.990	0.320
Department (internal medicine ref. others)	0.680	0.389	1.750	0.081	−0.904	0.519	−1.740	0.082
Department (surgery ref. others)	0.527	0.380	1.380	0.167	−0.585	0.507	−1.160	0.248
Department (obstetrics and gynecology ref. others)	0.765	0.448	1.710	0.088	0.617	0.597	1.030	0.302
Department (pediatrics ref. others)	0.505	0.477	1.060	0.290	0.484	0.634	0.760	0.445
Department (emergency and ICU ref. others)	0.888	0.474	1.870	0.062	−0.336	0.632	−0.530	0.595
Night shift (5–8 ref. < 5)	−0.328	0.241	−1.360	0.174	−0.315	0.322	−0.980	0.328
Night shift (> 8 ref. < 5)	−0.433	0.301	−1.440	0.151	0.116	0.401	0.290	0.772
Work years (6–10 ref. < 1–5)	0.173	0.298	0.580	0.561	0.492	0.397	1.240	0.216
Work years (11–15 ref. < 1–5)	−0.301	0.415	−0.720	0.469	1.099	0.552	1.990	0.047
Work years (≥ 16 ref. < 1–5)	−1.175	0.549	−2.140	0.033	1.746	0.733	2.380	0.017
Monthly income (3,001–6,000 ref. ≤ 3,000)	−0.430	0.403	−1.070	0.286	−1.021	0.537	−1.900	0.058
Monthly income (6,001–10000 ref. ≤ 3,000)	−0.661	0.439	−1.500	0.133	−0.646	0.586	−1.100	0.271
Monthly income (≥ 10,001 ref. ≤ 3,000)	−0.635	0.524	−1.210	0.226	0.006	0.697	0.010	0.993
Intercept	3.586	0.953	3.760	< 0.001	15.187	1.280	11.860	< 0.001
*R* ^2^	0.653				0.253			

To analyze the impact of authentic leadership and organizational climate on job embeddedness through affective commitment, we used 5,000 bootstrap samples to test the direct and indirect effects of the mediating model. The results showed that the direct impact of authentic leadership on job embeddedness is significant [*b* = 0.054, 95% CI = (0.013, 0.096)], which was consistent with Hypothesis 1. Meanwhile, authentic leadership also had a significant indirect impact on job embeddedness through affective commitment [*b* = 0.068, 95% CI = (0.041, 0.096)]. Therefore, Hypothesis 3 was supported. Furthermore, the results showed that organizational climate only indirectly and significantly affects job embeddedness through affective commitment [*b* = 0.116, 95% CI = (0.066, 0.167)]. Therefore, Hypothesis 2 was not supported. The hypothesis that affective commitment mediates between organizational climate and job embeddedness (Hypothesis 4) was supported. Table 4 shows the direct and indirect effects between the variables.

**TABLE 4:** Direct and indirect effects with 95% confidence intervals (CI).

Pathways	B	SE	95% CI	
			Lower	Upper
**Direct effect**				
Authentic leadership→job embeddedness	0.054	0.021	0.013	0.096
Organizational climate→job embeddedness	0.056	0.031	−0.006	0.117
**Indirect effect**				
Authentic leadership→affective commitment→job embeddedness	0.068	0.014	0.041	0.096
Organizational climate→affective commitment→job embeddedness	0.116	0.026	0.066	0.167

## Discussion

Since job embeddedness provides effective nurse retention strategies, more attention has been paid to leadership and organizational factors affecting nurse job embeddedness in recent years, such as authentic leadership and organizational climate. However, there is no research exploring the mechanism of the combined effect of the two variables on nurse job embeddedness. Taking Chinese nurses as a sample, this study found that affective commitment played a partial mediating role in the effect of authentic leadership on nurses’ job embeddedness and fully mediated the effect of organizational climate on nurses’ job embeddedness. Current findings provide nuanced insights into how authentic leadership and organizational climate influence nurses’ job embeddedness, moving beyond the conventional focus on mere correlations between leadership, organizational factors, and job embeddedness in prior research.

### Authentic leadership and job embeddedness

The current analysis results indicated a positive correlation between authentic leadership and nurses’ job embeddedness, meaning that when nurses perceive that their leaders exhibit higher authentic leadership traits, their job embeddedness also increases accordingly. This research emphasizes the significant impact of leadership style on nurses’ work experience, which is consistent with previous research findings (Cho et al., 2019; Song et al., 2024b). Authentic leaders establish high-quality leader–member exchanges through their inherent moral values, transparency, and balanced decision-making (Stewart Wherry, 2012). Meanwhile, as a relational leadership style, authentic leadership is more effective in creating a supportive work environment compared to a task-oriented leadership style (Cummings et al., 2021). Research by Harris et al. (2011) has suggested that high-quality leader–member exchanges and leader support are important factors in promoting individual job embeddedness. Additionally, authentic leadership has been proven to be beneficial in reducing workplace uncertainty and increasing subordinates’ psychological security (Alilyyani et al., 2018), which is particularly important for nurses who are exposed to high-intensity work and potential workplace violence. Simultaneously, according to the job demands–resources model (JD-R), the perceived authentic leadership of subordinates can help nurses cope with potential job demands, alleviate work pressure, and enhance their job embeddedness (Song et al., 2024b).

### The mediating role of affective commitment between authentic leadership and job embeddedness

In addition to the direct impact of authentic leadership on job embeddedness, affective commitment plays a mediating role between the two variables. The perception of authentic leadership can enhance the affective commitment level of nurses, which, in turn, positively impacts job embeddedness. Previous studies have shown that the moral qualities and behavioral approaches possessed by authentic leaders, such as integrity, honest behavior, transparent and cooperative relationships with followers, and balanced processing of information, can effectively cultivate subordinates’ trust and identification with leaders in the workplace (Ribeiro et al., 2018; Walumbwa et al., 2008). The establishment of trust and identification can effectively drive followers (Walumbwa et al., 2008), making them willing to exert more effort to achieve organizational goals, which enhances their sense of belonging and mission to the organization and their emotional connection to their current organizational position. This finding is consistent with SET. In addition, authentic leaders also promote the establishment of positive interpersonal relationships and trust among members of the team. In such a positive work environment, emotional connections between employees deepen (Rego et al., 2013; Ribeiro et al., 2018). Clinical nursing work is often built on cooperation. In a harmonious department team, nursing members help and trust each other, which is conducive to achieving greater work performance. However, leaving involves higher hidden costs, so authentic leadership enhances nurses’ affective commitment and, ultimately, deepens job embeddedness.

### Organizational climate and job embeddedness

Furthermore, this study found that organizational climate did not have a significant direct effect on nurses’ job embeddedness. This result is inconsistent with previous research findings (Hashim et al., 2015; Song et al., 2024a). The reason may lie in the differences in the samples. For example, Hashim et al. (2015) analyzed a sample of 114 disabled employees in private institutions. On the other hand, Song et al. (2024a) only included young nurses and did not consider the impact of demographic factors in the analysis. Regarding demographic factors, this study confirmed the significant predictive effect of work experience and employment methods on job embeddedness. The consideration of these control variables is beneficial for revealing the true relationship between organizational climate and job embeddedness more clearly. Moreover, the external environmental factors faced by nurses, the complexity of job embeddedness, and the social characteristics of high power distance in China can also provide some explanation for the current results. Specifically, the high work pressure faced by nurses (Park and Woo, 2018) and external environmental factors such as institutional measures in hospitals and the healthcare industry may have a more direct impact on nurses’ job embeddedness than organizational climate. Second, due to the complexity of job embeddedness, the intrinsic motivation of nurses themselves, such as professional self-concept (Goliroshan et al., 2021) and job meaning (Park and Woo, 2018), are also important factors affecting their embeddedness. The impact of these factors may outweigh the role of organizational climate. Meanwhile, in Chinese society with high power distance, subordinates are more willing to choose obedience (Yang et al., 2020), so nurses often pay more attention to the authority of superiors and underestimate the influence of organizational climate.

### The mediating role of affective commitment between organizational climate and job embeddedness

Moreover, the study found that affective commitment completely mediated the impact of organizational climate on job embeddedness. This finding indicates that a positive organizational climate can enhance nurses’ affective commitment, thereby promoting their engagement and sense of identification in their work. Organizational climate represents the shared perceptions and feelings of members toward the basic elements of the organization, which reflect the values and established norms of organizational culture (Emmanuel et al., 2020). Affective commitment is more closely related to intangible or abstract elements in the organization such as culture and values (Yang et al., 2019), providing support for the connection between organizational climate and affective commitment. Employees with high affective commitment exhibit higher job satisfaction (Saha and Kumar, 2018), loyalty to the organization (Zhao et al., 2013), and a sense of belonging (Meyer et al., 2004), which are factors that hinder employees from leaving their jobs. For nurses, being in a supportive organizational climate makes it easier to perceive the potential positive culture and values of the department (Ahmed et al., 2019; Ying et al., 2007) and enhances their identification and attachment to the department. This sense of identification will encourage nurses to invest more emotions and energy into their work in return, enhancing their sense of responsibility and belonging toward their jobs. Therefore, the organizational climate further enhances the job embeddedness of nurses through its direct impact on affective commitment.

### Implications

The current research has implications for nursing management. First, managers of medical institutions should be aware that the authenticity of leadership can enhance the level of job embeddedness among nurses. Managers, especially head nurses, should strive to change their leadership style and actively cultivate authentic leadership. For example, leaders should establish a sound platform for interactive communication with subordinate nurses, provide them with bottom-up feedback channels for opinions and suggestions, and maintain the smooth transmission of information. At the same time, leaders should share information with nurses, be receptive to different opinions and ideas, and provide timely feedback on nurses’ suggestions. Second, authentic leadership can also enhance nurses’ affective commitment, which conveys the positive impact of authentic leadership on job embeddedness. Leaders should set an example for nurses, act according to values and moral beliefs, enhance subordinates’ emotional attachment and identification with work and the organization, make subordinates loyal followers, and deepen their connection with the organization. Third, improving the organizational climate may be a valuable strategy to enhance nurses’ affective commitment, thereby increasing their level of job embeddedness. Managers should strive to create a positive organizational climate and culture in the department by fostering team communication, collaboration, and trust to strengthen the emotional connection between nurses and the organization, thereby enhancing job embeddedness. Additionally, nursing managers should enhance nurses’ sense of identity, loyalty, and sense of belonging to the organization through incentives and career development opportunities.

### Limitations

This study also has some limitations. First, the participants in this study were all clinical nurses from China, which may limit the generalizability and popularization of the results in other cultural contexts. Given the cultural differences in job embeddedness (Rafiq et al., 2022), future research should be conducted in different cultural contexts to gain a more comprehensive understanding of job embeddedness among nurses. Second, the data in this study were collected through self-reporting by participants, which may have resulted in recall bias. Third, because this study was cross-sectional, it was not possible to determine the causal link between authentic leadership, organizational climate, affective commitment, and job embeddedness. Therefore, more longitudinal studies are needed to determine the causal relationships between the aforementioned variables.

## Conclusion

This study investigated the impact of authentic leadership, organizational climate, and affective commitment on job embeddedness among Chinese nurses. The results indicate that authentic leadership has a direct and significant impact on nurses’ job embeddedness, while organizational climate has no significant effect on job embeddedness. Meanwhile, affective commitment partially mediates the relationship between authentic leadership and job embeddedness and fully mediates the impact of organizational climate on job embeddedness. This study contributes to the existing literature by clarifying the mechanisms through which leadership and contextual factors influence retention-related outcomes in a non-Western, high-power distance cultural setting. It underscores the critical mediating role of affective commitment, suggesting that future theoretical models should incorporate emotional attachment as a key process variable linking organizational contexts to employee stability. From a practical perspective, healthcare organizations and nursing managers nursing managers should focus on cultivating and developing authentic leadership styles and a positive organizational climate, which are effective ways to enhance the job embeddedness of nurses.

## Data Availability

The raw data supporting the conclusions of this article will be made available by the authors, without undue reservation.
